# Unlocking the potential of serious games for rehabilitation in low and middle-income countries: addressing potential and current limitations

**DOI:** 10.3389/fdgth.2025.1505717

**Published:** 2025-01-31

**Authors:** Diriba Dereje, Dheeraj Lamba, Teklu Gemechu Abessa, Chala Kenea, Cintia Ramari, Muhammad Osama, Oyéné Kossi, Paul Muteb Boma, Jules Panda, Anna Kushnir, Joanna Mourad, Jean Mapinduzi, Maryam Fourtassi, Kim Daniels, Judith Deutsch, Bruno Bonnechère

**Affiliations:** ^1^ Department of Biomedical Sciences, Faculty of Medical Sciences, Institute of Health, Jimma University, Jimma, Ethiopia; ^2^ REVAL Rehabilitation Research Center, Faculty of Rehabilitation Sciences, Hasselt University, Diepenbeek, Belgium; ^3^ Department of Physiotherapy, Faculty of Medical Sciences, Institute of Health, Jimma University, Jimma, Ethiopia; ^4^ Department of Special Needs and Inclusive Education, Jimma University, Jimma, Ethiopia; ^5^ Department of Information Science, Faculty of Computing and Informatics, Jimma Institute of Technology, Jimma University, Jimma, Ethiopia; ^6^ BCTRIMS, Brazilian Committee for Treatment and Research in Multiple Sclerosis, Belo Horizonte, Brazil; ^7^ Foundation University College of Physical Therapy, Foundation University Islamabad, Islamabad, Pakistan; ^8^ Brainstorm Research, Islamabad, Pakistan; ^9^ ENATSE, Parakou, Benin; ^10^ Virtual Rehabilitation Center of Lubumbashi, Institut de Recherche en Science de la Santé, Lubumbashi, Democratic Republic of Congo; ^11^ Reference Centre for Sickle Cell Disease of Lubumbashi, Institut de Recherche en Science de la Santé, Lubumbashi, Democratic Republic of Congo; ^12^ Department of Surgery, Faculty of Medicine, University of Lubumbashi, Lubumbashi, Democratic Republic of Congo; ^13^ Elita Rehabilitation Center, Lviv, Ukraine; ^14^ Psychomotor Therapy Institute, Saint-Joseph University of Beirut, Beirut, Lebanon; ^15^ Department of Physiotherapy and Rehabilitation, Department of Clinical Sciences, National Institute of Public Health (INSP), Bujumbura, Burundi; ^16^ Laboratory of Life and Health Sciences, Faculty of Medicine and Pharmacy of Tangier, Abdelmalek Essaadi University, Tétouan, Morocco; ^17^ Department of PXL—Healthcare, PXL University of Applied Sciences and Arts, Hasselt, Belgium; ^18^ Rutgers School of Health Professions, Newark, NJ, United States; ^19^ Technology-Supported and Data-Driven Rehabilitation, Data Sciences Institute, Hasselt University, Diepenbeek, Belgium

**Keywords:** serious games, rehabilitation, low and middle-income countries, healthcare access, technology, gamification, digital health

## 1 Introduction

In low and middle-income countries (LMICs), the burden of disability and the need for rehabilitation services are substantial (1). Rehabilitation encompasses a wide array of interventions and is defined by the World Health Organization (WHO) as “a set of interventions designed to optimize functioning and reduce disability in individuals with health conditions in interaction with their environment” (2). According to the WHO, an estimated 2.4 billion people globally are in need of rehabilitation, with a significant portion residing in LMICs (3). These countries indeed face a variety of challenges due to pervasive poverty, limited healthcare infrastructure, and constrained resources, which contribute to the high prevalence of disability and limited access to necessary rehabilitation services (4). Disabilities not only arise from congenital conditions and non-communicable diseases but are also exacerbated by external factors such as armed conflicts and inadequate medical facilities (5). Therefore, the need for rehabilitation is expected to rise due to population aging and the increasing prevalence of chronic conditions, making access to quality rehabilitation services more critical than ever (3), as the WHO projects that non-communicable diseases will account for 80% of the disease burden in LMICs by 2030 (6). Additionally, demographic shifts show rapid population aging in these regions, with estimates suggesting a 200% increase in older populations by 2050 (7). These trends, combined with high rates of trauma and injury, create an urgent need for accessible rehabilitation services.

The integration of rehabilitation into health systems can improve quality of life, reduce healthcare costs, and enhance workforce productivity (8). Studies have indeed demonstrated that every dollar invested in rehabilitation service yields a 9–11 return through reduced healthcare costs and improved workforce participation (9). Research shows that integrated rehabilitation programs reduce hospital readmission rates by up to 30% and improve patients’ functional outcomes by 40%–60% (10). Nevertheless, LMICs often face significant challenges in providing equitable access to quality rehabilitation care (2). These countries indeed often face a multitude of obstacles, including limited resources, infrastructure constraints, and geographical barriers, all of which impede their ability to provide equitable access to high-quality rehabilitation care (11). The most critical points are the shortage of rehabilitation professionals (with ratios as low as 0.5 therapists per 10,000 population in some regions), limited infrastructure (particularly in rural areas) (12), and financial barriers where out-of-pocket expenses can exceed 40% of household income (13). These factors further exacerbate the already substantial burden of rehabilitation needs in these regions (14).

In recent years, digital technology has demonstrated significant potential for delivering public health, health systems and health interventions remotely in LMICs (15). Particularly, serious mobile games and open-source technologies have promising evidence in supporting improved efficacy of public health solutions in LMIC settings (16). Serious games are a form of interactive digital media designed for a specific purpose beyond mere entertainment (17). They are specifically “games that do not have entertainment, enjoyment, or fun as their primary purpose.” Serious games aim to achieve objectives such as education, training, human resource management, and health improvement (18). These games encompass interactive computer applications that may include extensive hardware components, offering users valuable skills, knowledge, or attitudes while remaining challenging, entertaining, and engaging. Serious games integrate therapeutic exercises with engaging game mechanics, such as challenges, rewards, and progress tracking, to motivate patients to participate actively in their rehabilitation programs. Have demonstrated considerable potential in improving rehabilitation outcomes in high-income countries (19). By integrating therapeutic exercises and engaging gameplay mechanics, serious games offer an innovative approach to rehabilitation that can potentially address the challenges faced by LMICs and help to overcome the shortage of rehabilitation specialists.

This paper seeks to explore the potential of serious games to bridge the gap in rehabilitation access in LMICs, particularly by offering cost-effective, accessible solutions to regions where conventional rehabilitation services may be unavailable or prohibitively expensive. Serious games have the potential to reduce the burden on healthcare systems by allowing patients to participate in home-based rehabilitation (20, 21), thereby alleviating the strain on limited healthcare facilities. Additionally, serious games can address barriers to rehabilitation adherence, as they offer engaging, user-friendly formats that increase motivation and engagement among patients. We will examine the potential of serious games for rehabilitation in LMICs, evaluating both their opportunities and limitations. By highlighting the current barriers and proposing strategies to overcome these challenges, we aim to demonstrate how serious games can be transformative tools for rehabilitation in LMICs. Furthermore, we will discuss how technological advancements, policy support, and stakeholder collaborations can contribute to making serious games an accessible and effective component of rehabilitation in LMICs, ultimately reducing healthcare disparities and improving outcomes for patients.

## 2 Potential of serious games in LMICs

Digital games can enhance rehabilitation by improving both quality and efficiency. They offer a welcome alternative to traditional methods, mitigating the potential for monotony and providing scalable therapeutic interventions. Serious games have several characteristics that make them particularly suitable for rehabilitation in LMICs. First, they can provide accessible and cost-effective rehabilitation solutions (22). In regions with limited healthcare resources, serious games can be deployed on low-cost devices such as smartphones or tablets, enabling remote access to rehabilitation programs (23). This not only expands the reach of rehabilitation services but also reduces the financial burden on individuals and healthcare systems, through the provision of home-based therapy and reduced therapist time per patient. Furthermore, their digital nature facilitates widespread distribution via the internet, enabling personalized experiences and accessibility in home and remote settings (24).

Second, serious games have the potential to enhance patient motivation and engagement, which is crucial for successful rehabilitation (17). By incorporating game elements such as rewards, challenges, and social interaction, serious games can make therapy sessions more enjoyable and encourage patients to adhere to their rehabilitation programs (25). This can be especially beneficial in LMICs where access to traditional rehabilitation centres might be limited, and patients may struggle with adherence due to various barriers (26).

Third, serious games can address cultural and contextual factors by offering localized content and incorporating culturally relevant narratives and characters (27). Cultural sensitivity is crucial in healthcare, and serious games can be designed to resonate with the values, beliefs, and practices of the target communities. This customization can enhance the acceptability and engagement of rehabilitation interventions in LMICs, making them more effective in addressing the specific needs of the population (28). Locally developed serious games offer sustainability and customization to cater to cultural preferences and specific patient needs. They can be adapted for various conditions and task-oriented training, helping patients transfer acquired skills to real-life daily tasks.

## 3 Current situation

To assess the current use of serious games in LMICs, we conducted a bibliometric analysis. This method quantitatively evaluates research trends and the impact of studies within a specific field (29). We systematically searched two major databases, Web of Science and Scopus, for publications related to serious games, rehabilitation, and LMICs. A total of 1,564 articles were included in the analysis, with data processed using VOSviewer to visualize and map collaboration networks (30).

**Figure 1:**
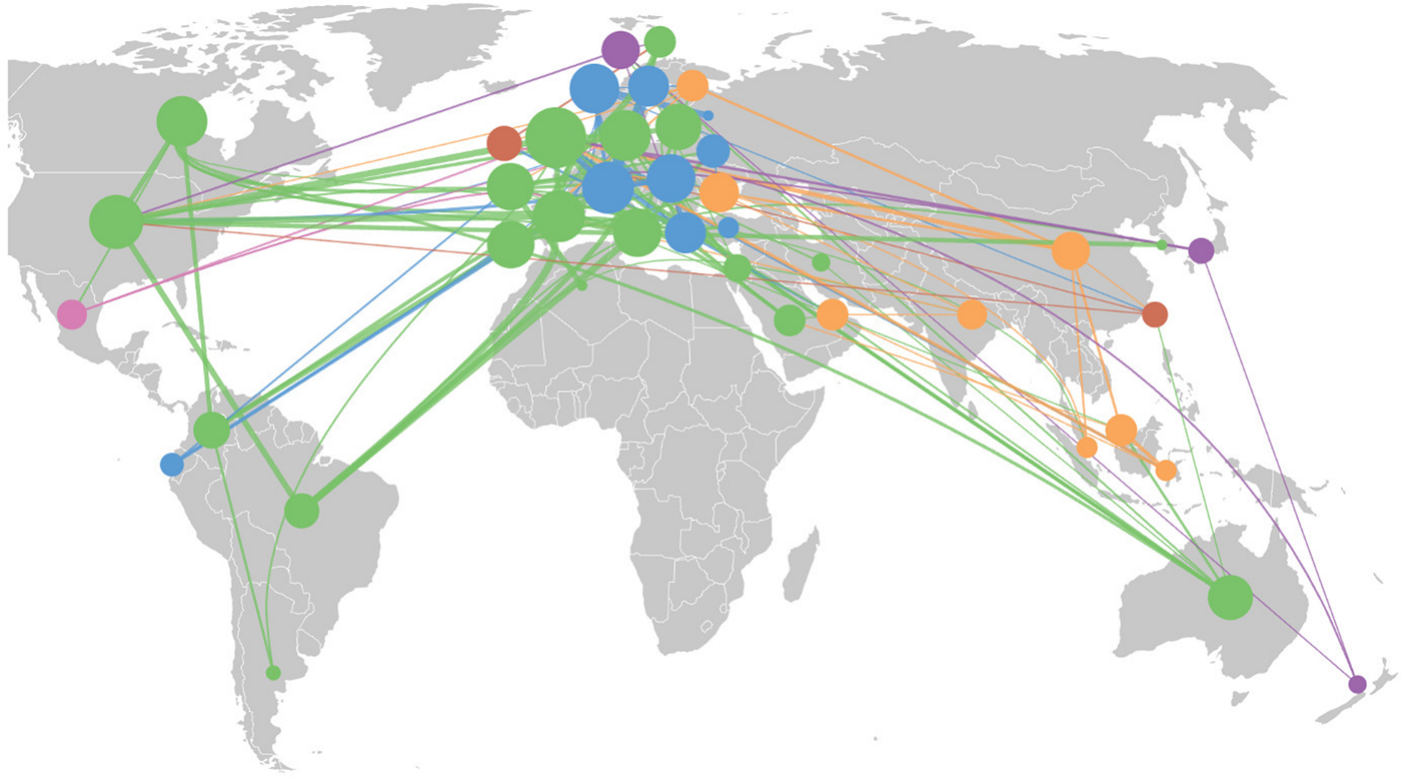
Geographical distribution map of collaborations. The size of each circle corresponds to the number of studies and collaborations.

Our analysis identified 88 countries with at least one author contributing to these studies. To ensure meaningful statistical analysis while maintaining representativeness, we established a threshold of 10 publications per country, resulting in 42 countries for further analysis. This threshold was chosen based on several considerations: (i) it provides a balanced trade-off between including a sufficient number of countries for robust comparative analysis while excluding those with limited research activity, (ii) it aligns with statistical requirements for minimum sample sizes in comparative studies (31, 32), and (iii) it captures approximately 90% of the total publication output while reducing noise from countries with sporadic research contributions. As shown in Figure 1, the retained countries formed significant research clusters in Europe, the Middle East, North America, and Australia. Unfortunately, the African continent shows minimal representation, with only Algeria contributing 12 publications.

However, different mHealth solutions have already been developed and implemented in Africa, mostly to provide relevant information to patients and increase health literacy (33): Hello Doctor (South Africa): advice and medical assistance; Mobile Widewife (Nigeria): voice messages sent to pregnant women for follow-up pregnancy; M-Pedigree (Kenya): drug identification; My Healthline (Cameroon): answers to questions on sexuality, family planning and HIV/AIDS; mHero (Liberia): information on the virus outbreak Ebola; Djobi (Mali, Senegal): mobile application contributing to reducing infant mortality and kindergarten in Senegal and Mali through mutual health insurance. These first experiences have shown the interest that SSA has regarding the use of mobile telephony for health actions. In rehabilitation *Captain Log's* has been used in Uganda to improve knowledge and cognition (34), *MyDailyRoutine*, is a serious game designed to assist patients with cerebral dysfunction, incorporating activities like virtual coffee preparation (35). *RehabCity* simulates a city environment, requiring users to engage in everyday tasks within its virtual streets, buildings, and parks (36). But commercial solutions (e.g., Nintendo Wii Fit) have also been successfully implemented to provide rehabilitation in low-income community in South-Africa (37). Other studies have also demonstrated the feasibility and a good adherence to rehabilitation program provided with the Microsoft Xbox Kinect in LMICs (38).

## 4 Limitations and challenges

**Figure 2:**
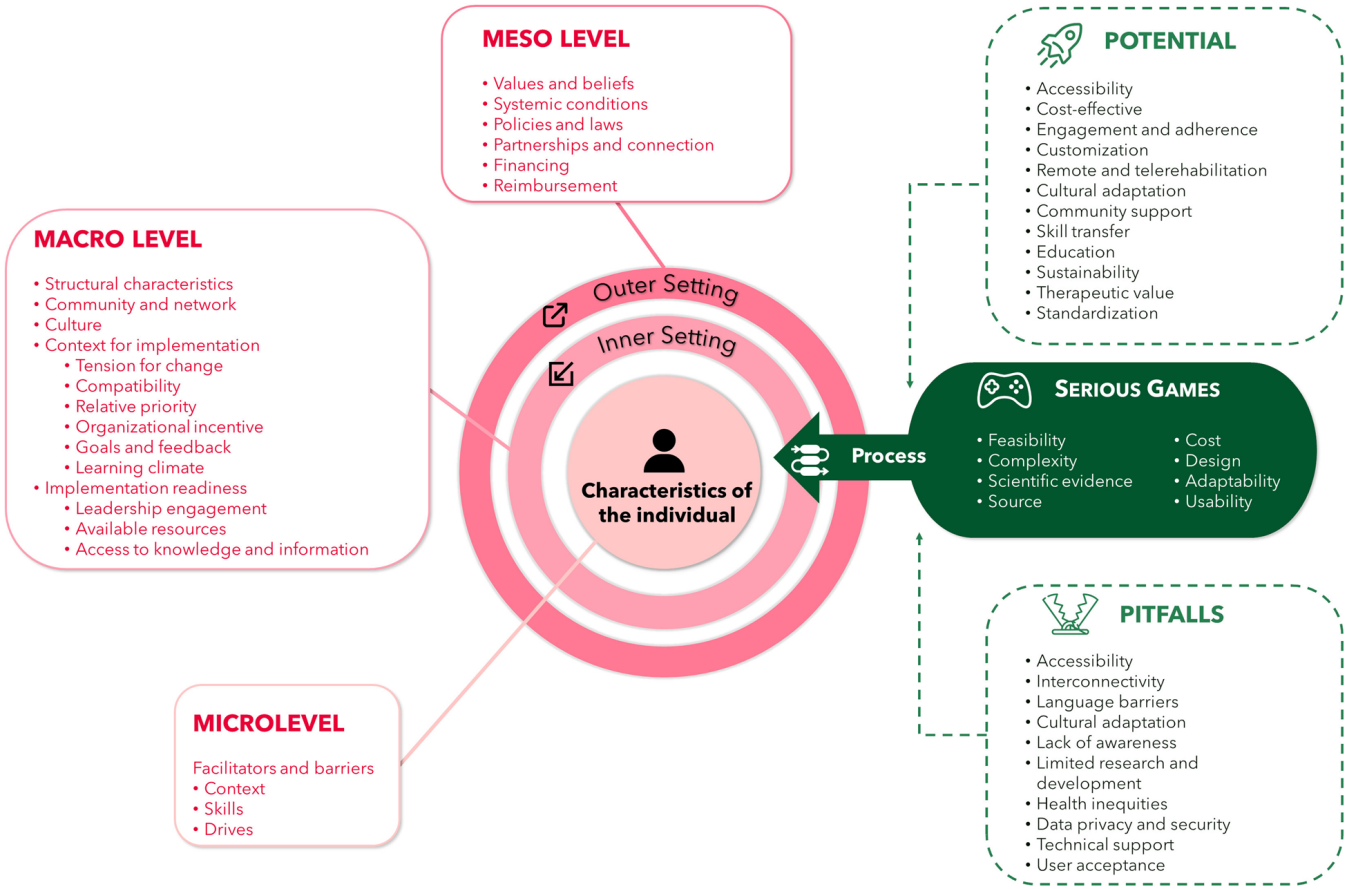
Consolidated framework for implementation research CFIR—implementing serious games in LMICs.

Despite their potential, serious games for rehabilitation in LMICs face various limitations and challenges that need to be addressed before taking full advantage of their benefits. Figure 2 outlines a consolidated framework for implementing serious games in LMICs, serving as a foundation for addressing current limitations and maximizing the effectiveness of rehabilitation strategies. This framework, based on an implementation research model, incorporates multilevel strategies to overcome the infrastructural, cultural, and educational barriers prevalent in these regions and illustrates the complex interplay between these domains and how they influence the successful implementation. It highlights that success depends not only on the technology itself but also on the broader ecosystem in which it is implemented. The adoption of serious games faces multiple barriers beyond technical issues. These include ethical considerations, policy limitations, administrative challenges, disparities in healthcare access, and insufficient resources. There are also concerns about the quality and reliability of research evidence. From a technical standpoint, key obstacles include disconnected systems that lack long-term sustainability, undefined industry standards, questionable data accuracy, limited technological infrastructure, and insufficient skilled personnel. To address these challenges, several approaches have been suggested: gaining support from policymakers, fostering partnerships across different sectors, increasing financial support, developing consistent regulatory guidelines, conducting flexible research studies, enhancing healthcare workers’ skills, and maintaining open dialogue with the public through clear communication channels (39).

More in details, one significant challenge is the lack of technological infrastructure, digital health literacy or skills and access to appropriate devices (40). Many LMICs struggle with limited internet connectivity, inadequate power supply, and outdated hardware, which can hinder the effective deployment of serious games of other portable technologies (41). However, Sub-Saharan Africa (SSA) has seen a surge in mobile phone, computer, and internet usage. The GSMA projects substantial growth in the SSA mobile market, forecasting a compound annual growth rate of 4.6% between 2019 and 2025, outpacing the global average of 3% (42). This rapid expansion positions SSA as one of the world's fastest-growing regions for mobile phone subscriptions.

However, investments in improving technological infrastructure is crucial, including initiatives to expand internet connectivity and provide affordable and suitable devices for game-based rehabilitation. Power generation shortages, leading to electricity load shedding and power cuts is also a common occurrence in LMICs, which can be a major barrier in the implementation of serious games for healthcare and rehabilitation purposes (43).

Additionally, the cultural context of LMICs may influence the design and content of serious games. Localization efforts must consider language barriers, cultural sensitivities, and the diversity of healthcare practices to ensure meaningful engagement and effective healthcare outcomes (44). One crucial note is that a significant portion of the population in LMICs may not be accustomed to technology, impacting user acceptance and adoption. Collaborating with local stakeholders, such as healthcare providers, patients, and community representatives, during the development of serious games can ensure their relevance and cultural sensitivity (45). This approach helps bridge the gap between inadequate or unavailable rehabilitative therapy and effective medical treatment.

Developing serious games in collaboration with local stakeholders, including healthcare providers, patients, and community representatives, can help ensure that the games are relevant and sensitive to the cultural context (45), this will ensure the medical transition between unsuitable and unavailable rehabilitative therapy as well as effective therapy.

However, integrating serious games into existing healthcare systems presents challenges, as it necessitates collaborative efforts among game developers, healthcare providers, policymakers, and local communities (46). This requires interdisciplinary cooperation and resource allocation, highlighting the obstacles faced in successfully implementing and sustaining serious games in rehabilitation practices (47).

## 5 Strategies to maximize effectiveness and accessibility

As digital health initiatives progress from the experimental phase to wider implementation, there is a growing emphasis on effective expansion and integration to offer lasting advantages to healthcare systems (Figure 2). To overcome the limitations and challenges, several strategies can be implemented. Insights from real-world case studies of serious games scaling in LMICs highlight five crucial focal areas for achieving success (48).

First, these programs or initiatives must possess inherent qualities that provide concrete solutions to unmet needs, incorporating input from end-users right from the beginning. Partnerships between game developers, rehabilitation specialists, and local stakeholders are essential to ensure the development of culturally appropriate and context-specific serious games (49). Collaborative efforts can also help leverage existing infrastructure, expertise, and resources to facilitate the deployment and evaluation of serious games (50). By involving all relevant stakeholders from the early stages of development, the games can be tailored to meet the specific needs and preferences of the target population (51).

Second, it is vital for all stakeholders to be actively engaged, well-trained, and motivated to support new implementations. Capacity building initiatives and the role of north-south training programs (52) can be implemented to enhance the skills of healthcare professionals in utilizing serious games for rehabilitation. This would empower local healthcare providers to effectively integrate serious games into their practice and maximize their potential impact. Training can include not only technical aspects of using the games but also understanding their potential benefits and limitations, as well as strategies to ensure patient compliance and engagement (53). At the WHO level, a rehabilitation competency framework has been developed to provide foundations for curricula for rehabilitation specialists (54). It is advocated to include serious games and new technologies related to rehabilitation in the competency framework.

Third, the technical design should prioritize simplicity, interoperability, and adaptability. Efforts should be made to improve technological infrastructure in LMICs, including expanding internet connectivity and ensuring access to affordable and suitable devices for game-based rehabilitation. Africa currently indeed faces the challenge of having the lowest internet penetration rate among continents, with 42% of its population having access to the internet (55). The introduction of the new technology necessitates a careful adjustment to the specific context, taking into consideration local physical barriers, particularly the availability of clinical facilities (56). Furthermore, it requires a conscientious adaptation to the existing lack of internet access, which involves advocating for offline applications and carefully limiting contacts between users and healthcare professionals. This may require collaboration with governments, non-governmental organization, and private sector entities to invest in and support the necessary infrastructure improvements.

The fourth focus is on the policy landscape, emphasizing the need for alignment with comprehensive healthcare policies and securing sustainable funding, including contributions from the private sector when applicable. From a public health perspective, it is imperative to engage in concerted and harmonized endeavours aimed at the successful integration of novel interventions into the healthcare system. These efforts span three distinct tiers: the macro level (57), encompassing legal, regulatory, and economic facets; the meso level (46), which pertains to local health services and community dynamics; and the micro level (50), which is intricately linked with patient-level considerations. The integration of multilevel models elucidating the interplay between immediate and distal determinants of health has markedly enriched our comprehension of the mechanisms underlying health disparities. Consequently, the inclusion of these multifaceted dimensions is of paramount significance in our analytical pursuits.

Lastly, consideration must be given to the external ecosystem, ensuring the availability of the necessary infrastructure to support large-scale digital initiative deployment. The development of local scientific research capability is a crucial undertaking, supported by the need to gather evidence that supports the effectiveness of innovative solutions (58). This necessitates careful examination of local and cultural nuances, in addition to technical limitations like inadequate infrastructure. Currently, research efforts focused on exploring emerging serious games advancements and assessing their effectiveness predominantly occur in countries with high-income economies. As a result, the practical implementation of these discoveries in LMICs presents complex difficulties, underscoring the need for prompt development of local scientific evidence.

It is imperative to determine the viability and acceptance of modified technology within the scope of both patients and professionals. Following this assessment, there is a need to determine the amount of evidence at the regional level, which is particularly relevant in the context of evidence-based practice. It is crucial to acknowledge that the rehabilitation goals in LMICs may differ from those in high-income nations, highlighting the need for research endeavours that are specific to the local context (59).

## 6 Conclusion

Serious games have the potential to revolutionize rehabilitation in LMICs by overcoming barriers to access and delivering cost-effective, engaging, and culturally relevant interventions. However, to unlock this potential, it is crucial to address the current limitations and challenges. By fostering collaborations, improving infrastructure, and promoting capacity building, serious games can become transformative tools that contribute to the improvement of rehabilitation outcomes and the reduction of healthcare disparities in LMICs.

Embracing this innovative approach can pave the way for a more inclusive and effective healthcare system, ensuring that all individuals have access to the rehabilitation services they need for a better quality of life. As technology continues to advance and more research is conducted on the effectiveness of serious games in LMICs, it is essential to remain adaptable and open to continuous improvement. By working together, researchers, developers, healthcare professionals, and policymakers can harness the potential of serious games to create a positive impact on rehabilitation and ultimately enhance the lives of individuals in LMICs.
